# Infertility care and the introduction of new reproductive technologies in poor resource settings

**DOI:** 10.1186/1477-7827-12-87

**Published:** 2014-09-08

**Authors:** Luis Bahamondes, Maria Y Makuch

**Affiliations:** Department of Obstetrics and Gynecology, School of Medical Sciences, University of Campinas (UNICAMP), Campinas, SP Brazil

**Keywords:** Infertility, Assisted reproductive technologies, Low cost, Inequity

## Abstract

**Background:**

The overall prevalence of infertility was estimated to be 3.5–16.7% in developing countries and 6.9–9.3% in developed countries. Furthermore, according to reports from some regions of sub-Saharan Africa, the prevalence rate is 30–40%. The consequences of infertility and how it affects the lives of women in poor-resource settings, particularly in developing countries, has become an important issue to be discussed in reproductive health. In some societies, the inability to fulfill the desire to have children makes life difficult for the infertile couple. In many regions, infertility is considered a tragedy that affects not only the infertile couple or woman, but the entire family.

**Methods:**

This is a position paper which encompasses a review of the needs of low-income infertile couples, mainly those living in developing countries, regarding access to infertility care, including ART and initiatives to provide ART at low or affordable cost. Information was gathered from the databases MEDLINE, CENTRAL, POPLINE, EMBASE, LILACS, and ICTRP with the key words: infertility, low income, assisted reproductive technologies, affordable cost, low cost.

**Results:**

There are few initiatives geared toward implementing ART procedures at low cost or at least at affordable cost in low-income populations. Nevertheless, from recent studies, possibilities have emerged for new low-cost initiatives that can help millions of couples to achieve the desire of having a biological child.

**Conclusions:**

It is necessary for healthcare professionals and policymakers to take into account these new initiatives in order to implement ART in resource-constrained settings.

## Background

The prevalence of infertility has been calculated as ranging from 4 to 14% worldwide, and the international consensus is that 8–10% of cohabiting couples are infertile, with variations in this percentage according to the region considered [1–4]. In 2002, in an analysis of data from demographic and health surveys in developing countries (excluding China), it was estimated that more than 186 million women who were married or in a stable union and of reproductive age presented primary or secondary infertility problems [5]. An analysis of 25 population-based surveys estimated the overall prevalence of infertility to be 3.5–16.7% in developing countries and 6.9–9.3% in developed countries [6, 7]. Furthermore, according to reports from some regions of sub-Saharan Africa, the prevalence rate is 30–40% [8].

It has also been estimated that around 5% of the world population decides voluntarily to be childless and does not choose the parental role as part of personal and adult development. Therefore, it may be legitimate to think that parenthood is socially expected and continues to be an important objective of the life project, considered part of personal development and adult life among most men and women in the world [9–16].

In a predominantly fertile world, it is expected that pregnancy will occur when men and women decide to become parents, and it is surprising when this does not happen. For most men and women, infertility is a disruption of the life project and a social situation that differs from the majority. Involuntary childlessness is considered a major life issue associated with psychological suffering and long-term consequences. Women who desire a child and cannot achieve pregnancy perceive themselves as different from women who are fertile, and feel they are losing something important in their lives [17–20]. The diagnosis of infertility may become a life crisis, and most couples need to develop mechanisms to cope with the temporary or permanent loss of fertility and the possibility of having a biological child [17, 21, 22]. After diagnosis, men and women have feelings of loss of control over their life project, and feel they are unable to achieve their life expectations [17, 18, 23–25].

Infertile couples may have difficulty communicating their feelings to family and friends with children because they do not perceive a comprehensive social environment to understand their situation [13, 26–28]. Frequently, they feel alone and without support to deal with the experience of infertility, and a need for sharing experience with other infertile couples, guidance through the treatment process, and written information about practical and emotional aspects of treatment to help them through the experience of infertility [29].

Over the last decades, many women have married or established stable relationships after achieving other life goals such as a career and financial stability, postponing the desire to have children. The mean age of women at the time of their first pregnancy has increased in recent years, and in the last decade was 28–29 years old in most of western hemisphere countries. In this scenario, many women try to become pregnant with their first child when fertility starts to decline and the risk of infertility is higher [30].

The consequences of infertility and how it affects the lives of women in poor-resource settings, particularly in developing countries, has become an important issue to be discussed in reproductive health. In some societies, the inability to fulfill the desire to have children, together with the social stigma associated with infertility, makes life difficult for the infertile couple. In many regions, infertility is considered a tragedy that affects not only the infertile couple or woman, but the entire family [13, 31, 32].

Many societies still view infertility as affecting only the women, and those who are childless are frequently neglected or exposed to humiliation and domestic violence [33–38]. Furthermore, this situation and their own suffering because of the impossibility of conceiving a child may lead to diverse psychological problems, such as distress, anxiety, depression, low self-esteem, feelings of blame and guilt, and reduced sexual interest [33, 37, 39–41].

Seeking for solutions, when there are no services available in the public sector or at an affordable cost, women may be induced to seek ineffective therapies [37, 39, 41]. Consequently, access to the diagnosis and treatment of infertility, including assisted reproductive technologies (ART), contributes to resolving social inequities and emotional difficulties. Infertility is more than a health problem; it is a social issue and a public health matter [42].

The treatment of infertility in low-resource settings is a challenge for policymakers and the health system. However, it is mainly a human rights issue: All men and women who desire to have children, to have a family, and not to be different from most of the individuals of their social environment should have the opportunity to solve this problem. The poorest sector of the populations of developing countries are probably those more prone to infertility due to poverty, poor education, early sexual debut, no access to services for counseling and treatment when necessary, unsafe abortion [37], among other factors. Probably, they represent the population most in need of ART [43–47].

More than 30 years have elapsed since the first publication reporting the birth of a child after *in vitro* fertilization (IVF) [48], much research has been done, and other techniques like intracytoplasmic sperm injection (ICSI) have emerged. ART has brought great hope to many couples, who have referred to this technique as their last chance of having a child that is biologically related to them [49].

Although ART is a common treatment for infertile couples worldwide, the availability of such procedures is lacking in developing countries; even in developed countries, low-income couples have great difficulties of access due to the high cost charged by private clinics and the lack of services offered by the public sector [45, 47, 50].

## Methods

This is a position paper which encompasses a review of the needs of low-income infertile couples, mainly those living in developing countries, regarding access to infertility care, including ART and initiatives to provide ART at low or affordable cost. Information was gathered from the databases MEDLINE, CENTRAL, POPLINE, EMBASE, LILACS, and ICTRP with the key words: infertility, low income, assisted reproductive technologies, affordable cost, low cost.

### Infertility: medical causes

Approximately one-third of infertility cases are due to the male factor, one-third to the female factor and the remaining third to a combination of male and female factors or to unidentified causes [51]. In settings with poor access to health services, common causes of infertility are post-partum and post-abortion infections, tuberculosis, and sexually transmitted infections (STIs) [52]. Infertility can also be a consequence of infections after female genital circumcision [53].

In some developing countries, cohabitation is a common practice and adolescent pregnancy rates are high. This early age of sexual debut may be related to a high prevalence of reproductive tract infection (RTI), and consequently both male and female infertility [2, 3, 6]. The prevalence of male and female RTI, and the infertility which may consequently occur, is high in developing countries [53, 54], and the best way to reduce this incidence is through prevention and RH education, an important task for governments; however, this represents a challenge [45–47], because education is a long-term process and individuals who are infertile because they do not use means of prevention need to be treated. It is well-known that tubal obstruction was the first diagnosis for which IVF was developed and indicated, and RTI often provokes tubal obstruction. The fact that this cause of infertility is more common in developing areas and among low-income populations is an indicator of the necessity of implementing ART in low-income populations.

### Access to infertility health care

As part of the United Nations (UN) Program of Action, consensus was reached on a comprehensive concept of RH that includes the right of men and women to choose the number, timing, and spacing of the children they desire to have. This Program of Action includes the need to incorporate family planning programs, the prevention and treatment of RTI, and the prevention and treatment of infertility as part of RH services [55]. Furthermore, the Millennium Developments Goals of the UN 2000 established universal access to RH as one of the targets to be achieved by 2015 [56]. Infertility is one of the neglected aspects of RH care, particularly in developing countries. In many developing countries, infertile couples with limited resources are confronted with difficulties and very restricted possibilities of gaining access to infertility services within the public health sector [26, 45–47].

The urgent need for many women to resolve their childlessness is a situation that increases the demand from poor couples in developing countries for good quality infertility care [33]. Delays in gaining access to the diagnosis of infertility, to infertility services and to services that offer ART may negatively affect the possibility and the success of treatment for many couples. Access to diagnosis and treatment for infertility, including ART procedures, contributes to diminishing inequities, and may reduce suffering related to the difficulty of access and emotional suffering due to infertility. This represents a step forward in guaranteeing the right of women and men to decide when they desire to have children and to help infertile couples to have at least one biological child [55, 56].

More than a decade ago, the World Health Organization (WHO) recommended that infertility must be considered a global health problem and also recommended the development of initiatives to improve access to infertility services and the care of infertile couples, including the development of low-cost ART [57]. Although this is still not sufficient and is far from representing a solution, some initiatives have been implemented to reduce the burden of infertility [7, 33, 57–60]. In this context, the European Society for Human Reproduction and Embryology (ESHRE) created a Task Force on Infertility and Developing Countries with the objective of explore new approaches to ART that will be useful in developing settings [61].

The nonexistence of infertility care in resource-constrained settings has been justified many times by the fact that other important and life-threatening health issues are a priority in the health sector, including maternal morbidity and mortality, vaccination, malaria, dengue fever, yellow fever, and the drugs required for people living with HIV and AIDS [54, 62, 63]. The implementation of infertility care, including affordable ART treatment, frequently represents a challenge; an understanding of the impact infertility has on the life of men, and particularly women, not only as a health problem but an emotional and social problem, as well as strong political commitment, is needed for action. According to the WHO [54], *‘relatively few of the world’s infertile men and women can be said to have complete and equitable access to the complete range of infertility treatments at affordable levels’.*

### Simplified procedures for ART

Many new initiatives to reduce the cost of ART procedures without hampering the results in terms of pregnancy rates and number of babies taken home have been developed over the last 10 years. However, despite the efforts to make these procedures available at affordable cost, efforts are still scarce; most of the procedures come from developed countries, and are still not translate into actions in many settings.

Many professionals working with ART procedures use controlled ovarian stimulation and follicle development with gonadotropin-releasing hormone (Gn-RH) analogues or antagonist and with gonadotropins in order to develop many ovarian follicles to obtain more embryos, and consequently have the possibility to transfer more than one embryo to maximize the possibility of pregnancy. However, in recent years, there have been many reports presenting reliable data on low-cost ART with acceptable pregnancy rates, and in some cases, rates that are similar to those obtained with high-cost procedures [64, 65].

Low cost does not necessarily jeopardize the quality of the procedures. Low-cost ART is based on the use of affordable stimulation protocols, clinical judgment rather than sophisticated laboratory testing, reduction or elimination of all superfluous pre-procedure investigations, careful use of disposable materials, and well-established protocols for laboratory routines. It has to be taken into account that the use of low-cost protocols does not avoid the necessity of the infrastructure of a good laboratory.

The use of the natural cycle or simplified ovarian stimulation ART treatment can reduce the cost of drugs, as well as the possibility of multiple pregnancies. However, to the best of our knowledge, there are no randomized clinical trials comparing natural cycle ART with standard ART. A recent review [66] showed that there is no evidence that clomiphene citrate (CC) versus gonadotropins and Gn-RH analogue or antagonists are equivalent in terms of follicular development. Nevertheless, CC is still the drug used in many protocols to reduce cost. It is well established that CC mimics the effects of Gn-RH analogue and prevents the luteinizing hormone (LH) surge [64].

A group of researchers [67] used CC (50 mg daily) with intermittent doses of human menopausal gonadotropin (hMG) 150 IU on alternate days from the 5th day onwards; follicular development was monitored only with pelvic ultrasound. The researchers performed embryo transfer for more than two-thirds of the women and showed that with this protocol, birth and clinical pregnancy rates per embryo transfer were similar to those expected with high-cost procedures. The average direct cost per cycle was US$ 675 for IVF and US$ 725 for an ICSI cycle.

Whether the use of low dose of CC in ART reduces premature ovulation rate and increases the transfer rate was also evaluated [68]. Women who underwent one natural-IVF cycle with human chorionic gonadotropin to induce ovulation were compared with women who underwent one natural-IVF cycle with 25 mg/day CC for almost 7 days. Women who used CC presented a significantly lower premature ovulation rate in comparison to those who did not use it and the transfer rate was higher among the CC group in comparison to women who did not use CC. Clinical pregnancy rates were not significantly different between groups.

Another low-cost strategy is the transfer of a single embryo. A Japanese-based study [69] assessed a cohort of more than 7,000 women who received a single-embryo transfer according to age (≤29, 30–34, 35–39, 40–44, and ≥ 45 years) who performed 20,244 cycles with a CC stimulation or natural-IVF cycle. Fertilization (80.3%) and cleavage (91.1%) rates were not significantly different among different age groups; however, overall live birth rate decreased as age increased, and was no higher than 1% in women aged 45 years or older. The results showed that single embryo transfer could be a strategy to reduce the cost of ART cycles.

Lopez-Regalado and co-workers [70] evaluated the pregnancy rate with single embryo transfer versus double embryo transfer among women under 38 years old. The cumulative live birth delivery rate in the single embryo transfer group was similar to the women who received double embryo transfer. Additionally, multiple gestations were significantly lower in the group who was treated with single embryo transfer than the other group (0% vs. 26.4%; *P* < .05). Rate of implantation, cumulative pregnancy rates per transfer, and cumulative live birth delivery were similar among both groups. Similar results were obtained in other settings [71, 72]. In a recent Cochrane review [73], the policy of single embryo transfer versus two embryo transfer was evaluated regarding pregnancy rates. The authors concluded that if a single fresh embryo is transferred, it is associated with a lower live birth rate than double embryo transfer. Nevertheless, they also observed no significant differences when single and double embryo transfer re compared regarding cumulative the live birth rate with repeated single embryo transfer, involving either two cycles of fresh single versus one cycle of fresh single embryo followed by one frozen single embryo in a natural or hormone-stimulated cycle. Furthermore, it was observed that single embryo transfer was linked to lower rates of multiple pregnancies which it is important in baby survival and cost of use of the intensive care unit. However, the evidence is related to young women without a poor prognosis.

Some years ago, a very low-cost ART procedure was reported in which the gametes and embryos were incubated in a capsule in the woman’s vagina, thereby avoiding the use of expensive and complex laboratory. This procedure resulted in adequate pregnancy rates of 19% per cycle [74].

In this vein, a recent case-series report [75] described the results of a pilot trial that involved a simplified laboratory method for human IVF. The described system reproduces the atmospheric and culture conditions for fertilization and pre-implantation embryogenesis, with no need for a culture chamber with gases. Using the described culture system, 8 out of 23 embryos implanted, one miscarried at eight weeks of gestation, and seven babies were born.

This simplified system could be incorporated worldwide to improve the capacity of offering ART at affordable cost in low-resource settings. There is no doubt that this strategy does not resolve all the issues involved in low-cost ART; however, it is a big step forward to help services to implement ART at affordable cost in low-income settings. The authors estimated that the first cost of a single IVF cycle using these methodologies and protocols must be less than 200 € [76].

### Comments

In developing countries and poor-resource settings, infertility care and treatment that includes access to ART at low cost or at least at affordable costs for the underprivileged segments of society is a neglected RH issue in public health in most countries. Services are mainly available in the private sector at a high cost for the majority of the population [33, 50, 61]. Frequently, health authorities and governments justify this neglected health issue and the lack of infertility services based on the fact that there are other urgent, life-threatening health problems to consider [55]. This suggests that many policymakers are not aware of the profound implications of infertility in the lives of individuals, couples, and families. Furthermore, in some settings, when unattended, this health issue may become a source of discrimination, abandonment, and violence, particularly for women.

Even in some settings in developed countries, for low-income populations like Latinos and African-American descendants in the United States (US) [77] or migrants in the European Union [78], infertility diagnosis and treatment including ART is a neglected RH issue. As an example of this inequity, a US survey [79, 80] evaluated more than 4,000 couples to assess the likelihood of seeking an infertility evaluation and infertility treatment. Among those seeking an evaluation, only 50% reported they had undergone any treatment. Low income, employment status, and non-white ethnicity were strongly correlated with the possibility of not seeking treatment; among those who received treatment, only a small proportion was treated with ART.

ART is an excellent solution for many infertile couples who cannot conceive the desired child by other treatments. High-quality ART procedures at no cost for patients or at an affordable cost to the underprivileged segment of the society is a moral and social obligation of governments that have promised to improve and provide RH services that are accessible to most sectors of the population [54]. Twenty years have elapsed from United Nations International Conference on Population and Development [56] and it is necessary to take initiatives to offer treatments to infertile couples that include ART procedures, improved access to the underprivileged population, and the guarantee of well-equipped and competently staffed infertility centers.

The lack of ART services at low or affordable cost increases the practice of couples seeking these procedures outside their country of residence. A new phenomenon of cross-border reproductive care can be observed worldwide, and many questions are emerging, mainly regarding whether patients traveling abroad for ART procedures are at any risk and if there is a really cost-benefit result. In addition, it is not clear whether the healthcare professionals (HCPs) who offer this kind of service advise potential patients of the real pregnancy rates, complications, offspring risks, or of the availability of gamete donors and surrogate mothers, among other thing [81].

The fact that there is a respectable body of research showing the effectiveness of low-cost technology with the aim of facilitating the inclusion of ART in restricted-resource settings does not guarantee the implementation of these strategies in infertility services. There seems to be a gap between the development of low-cost ART technology and the use of these strategies in a clinical context. The results of recent research on the new low-cost and simplified ART system for culturing gametes without the needs of a sophisticated laboratory [76] are encouraging, because this work addresses a fundamental obstacle for millions of couples worldwide, namely access to ART at very low or at least affordable cost [77, 82]. It is obvious that further studies are needed to validate these early results and to replicate them in different settings. As stated, it is necessary to evaluate the real cost, mainly in relation to hidden costs (personnel and infrastructure) [82]; however, any reduction in cost will be a great step for many couples.

The introduction of these technologies requires a specialized, organized medical and paramedical staff; a minimum of infrastructure within the health system—regarding the supply of materials and improvement in existing services—is necessary. Moreover, patients and staff need to interact with a cooperative understanding. HCPs need to explain clearly the specific characteristics of the treatment, the real possibilities of pregnancy, and potential risks to their patients, and must respect cultural and religious beliefs. Patients, on the other hand, need to follow the medication scheme and the intricate rules of these procedures carefully.

Given the magnitude of the problem—the number of people all over the world who suffer from infertility, the impact infertility has on people’s lives, and that ART is presently out of the reach for the majority of those who need it—it is legitimate to question the extent to which initiatives have to be carried out for these procedures to become part of national infertility policies. This is an issue for each country to resolve. However, as Fathalla *et al.* [83] stated almost a decade ago, it is time to cross the boundary from talking and writing to taking action.
